# Corrigendum to “Improvement of Memory by Means of Ultra-Low Doses of Antibodies to S-100B Antigen”

**DOI:** 10.1155/2020/3278048

**Published:** 2020-12-29

**Authors:** Evidence-Based Complementary and Alternative Medicine

In the article titled “Improvement of Memory by Means of Ultra-Low Doses of Antibodies to S-100B Antigen” [1], conflicts of interest should have been declared as follows. At the time of publication by OUP, the journal considered research on homeopathy, but no longer does.
